# Home electricity data generator (HEDGE): An open-access tool for the generation of electric vehicle, residential demand, and PV generation profiles

**DOI:** 10.1016/j.mex.2024.102618

**Published:** 2024-02-16

**Authors:** Flora Charbonnier, Thomas Morstyn, Malcolm McCulloch

**Affiliations:** Department of Engineering Science, University of Oxford, UK

**Keywords:** Home Electricity Data Generator (HEDGE), Datasets, Data-driven methods, Open access, Demand-side response, Distributed energy resources, Buildings, Smart grid, Residential

## Abstract

In this paper, we present the Home Electricity Data Generator (HEDGE), an open-access tool for the random generation of realistic residential energy data. HEDGE generates realistic daily profiles of residential PV generation, household electric loads, and electric vehicle consumption and at-home availability, based on real-life UK datasets. The lack of usable data is a major hurdle for research on residential distributed energy resources characterisation and coordination, especially when using data-driven methods such as machine learning-based forecasting and reinforcement learning-based control. We fill this gap with the open-access HEDGE tool which generates data sequences of energy data for several days in a way that is consistent for single homes, both in terms of profile magnitude and behavioural clusters.

• From raw datasets, pre-processing steps are conducted, including filling in incomplete data sequences, and clustering profiles into behaviour clusters. Transitions between successive behaviour clusters and profiles magnitudes are characterised.
• Generative adversarial networks (GANs) are then trained to generate realistic synthetic data representative of each behaviour groups consistent with real-life behavioural and physical patterns.
• Using the characterisation of behaviour cluster and profile magnitude transitions, and the GAN-based profiles generator, a Markov chain mechanism can generate realistic energy data for successive days.

## Graphical abstract

**Figure:**
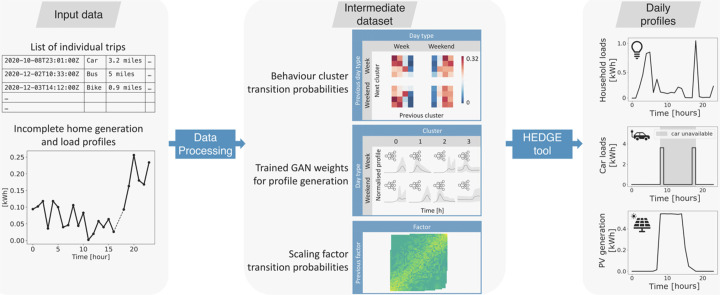

Specifications table

**Table:** 

Subject area:	Energy
More specific subject area:	Energy data
Name of your method:	Home Electricity Data Generator (HEDGE)
Name and reference of original method:	N/A
Resource availability:	Input data: • Customer-led network revolution [1,2] • National travel survey [3] Data preparation code, and Home Electricity Data Generator (HEDGE): https://github.com/floracharbo/hedge

**Method details**

## Objectives and motivation

The Home Energy Data Generator (HEDGE) tool tackles the challenge of how to generate home energy consumption and generation data for use in data-driven algorithms. This open-access tool^1^ can generate realistic photovoltaic (PV) generation, household loads, and electric vehicle (EV) consumption and at-home availability profiles.

The characterisation and simulation of residential energy resources is of increasing interest given their potential for demand-side response [4]. Renewable energy could supply 70% to 85% of electricity globally by 2050 in 1.5°C-compatible pathways^2^ [5], with corresponding needs for storage and demand-side response [4]. Demand-side response is also critical for the electrification of residential heating, cooling and transport, which, without coordination, could cause a significant increase in peak electricity demand with adverse consequences for low-voltage distribution networks [6]. Residential consumers could play an essential role in providing demand-side response [7], given as much as 53% of household demand could be flexible in the future [8].

**Fig. 1:**
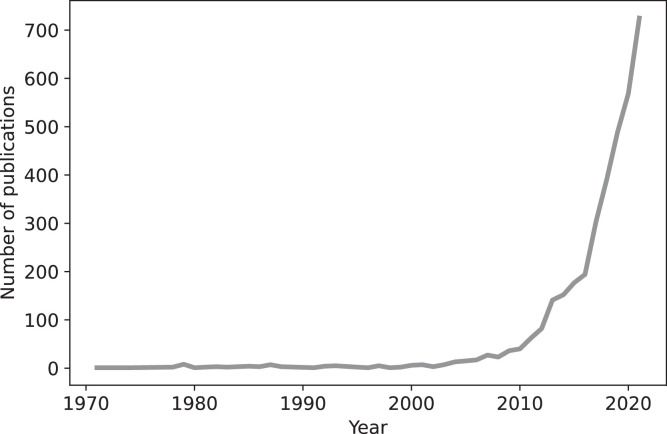
Number of publications of data-driven methods for residential energy.^3^

Data-driven methods are of particular interest in the field of residential energy forecasting [9] and control [10] for three main reasons. Firstly, there is high uncertainty at the local level [11], due to the small scale of residential electricity consumption and generation, and their behavioural and weather dependencies. Secondly, there are limitations to personal data sharing, particularly in realtime. This is due to both the limited availability of communication and computation infrastructure at the scale of individual homes and to the privacy requirements of the residential sector [12]. Thirdly, centralised optimisation methods have limited scalability [13,14]. Therefore, data-driven analysis and control of the residential energy sector are of increasing interest [10,13]. Fig. 1 thus shows that the number of publications in the field has increased exponentially since 2000 (30% average yearly increase).

A major hurdle for the development and implementation of such data-driven methods is the availability of large datasets on EV consumption and at-home availability, PV generation, and household consumption for training and testing data. The quality of data determines the results of data-driven methods such as machine learning (ML) predictions or reinforcement learning (RL) policies [15], and should be as much of a focus as algorithm selection. While large amounts of residential energy data are indeed collected, training directly on available data is often unsatisfactory given:

• The privacy and costs constraints of data collection, or cost of access to datasets that are not freely available without a licence or privileged access. Obtaining energy data frequently poses a significant challenge for the development of energy communities [16]. This can result in substantial time and financial resources being expended. Generally, open-access databases offer rather restricted access to comprehensive energy consumption and production profiles, as the establishment of open-access data initiatives is fraught with numerous legal and occasionally ethical obstacles and inquiries. Companies may be wary about sharing their energy data outside of their business [16].
• The limited number of years of data collection available (e.g., for electric cars, for which we only have smart trial data from early adopters), or the limited number of subsequent days of data available for a given household, which hinders consistent simulation of a home for more extended. For example, the National Travel Survey offers at most a week of travel data for a given household [3].
• The labour-intensiveness of pre-processing of data, with efforts repeated across individual projects, as datasets are often not in a usable format, or not self-consistent across different days. Data quality has thus been identified as a challenge for the adoption of AI in the smart energy industry [17]. A major hurdle identified by energy community initiators is thus that of data formatting standards and the quality of the acquired data [16].

While agent-based modelling approaches have been previously adopted to model residential data such as EV patterns [18], training data should reflect real-life resource intermittency and behaviour variability to minimise training losses in a robust way without over-fitting [19]. Purely synthetic data often lacks these characteristics [20]. Moreover, bottom-up models such as CREST [14] rely on assumptions on dwelling activities and thermal-electrical demand modes for generating data.

Therefore, novel methods are required to meet the needs of both large-scale datasets and the inclusion of real-life patterns. A standard residential energy data generation tool that could interface with a local energy system benchmarking environment to generate continuous daily energy data for several days in a consistent manner, both in terms of profile magnitude and behavioural clusters, would greatly benefit the research community.

We bridge this gap by proposing a new tool which generates EV, PV and household demand-related data semi-randomly based on large-scale real-life datasets, while preserving profile magnitude and behavioural consistency over time. Compared to [21], which first proposed the use of generative adversarial networks to generate smart grid-related data, we further provide a data generator for UK data, integrate this tool directly into a MARL benchmarking framework, and include EV data generation. While this model uses UK data, the model could be adapted to use similar data from other countries, so long as banks of data of household consumption, PV generation, and travel patterns are available.

**Fig. 2:**
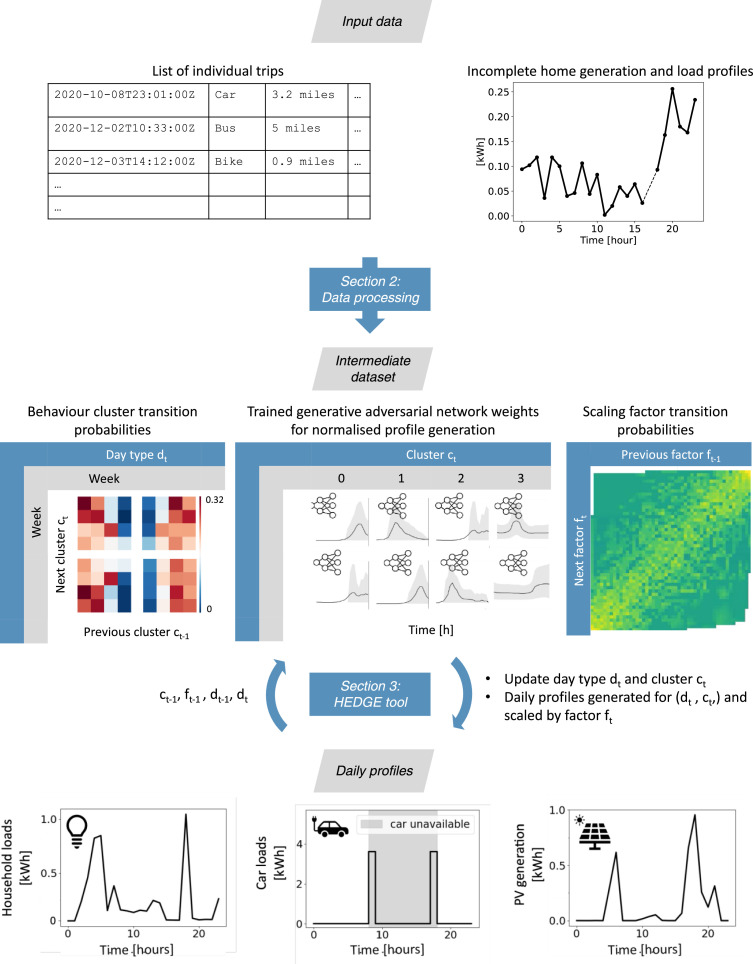
Workflow from raw input data to the generation of random realistic household energy profiles. The two main steps “Data processing” and “HEDGE tool” use correspond to the sections 2 and 3 of this MethodX document.

The rest of this MethodX paper is structured as illustrated in Fig. 2. In Section 2, we present the data pre-processing steps to obtain the intermediate data used by the HEGE tool. In Section 3, we then present the mechanism used by HEDGE to generate data profiles. Finally, we comment on the privacy benefits of the methodology presented in Section 4.

## Data preparation

The data preparation steps are listed in Table 1 and detailed in the subsections below.

**Table 1:** Data preparation steps.

Step	Solar generation	Household loads	Electric vehicles
1. Import data sources	Customer-led network revolution (CLNR) dataset TC1a	CLNR dataset TC5	UK National Travel Survey
2. Data selection and filtering	Only residential data is used, and for valid date ranges.		Only residential car journeys are selected.
3. Conversion to relevant daily profiles	Convert to resolution specified (which has to be greater than or equal to 1 min)	Get resolution specified (greater than or equal to 30 mins)	Convert list of trips to the distance travelled per time interval at the resolution specified. Infer the type of trip (motorway, urban, rural) from the location and distance. Then convert the distance travelled to electricity consumption based on the trip type. Infer at-home availability of the car based on trip times, origins and destinations.
4. Missing data interpolation	Linearly fill in single missing time steps or discard the day of data.		
5. Normalisation	Normalise daily profiles by the sum of the electricity consumption/generation over one day. Record the scaling factors.		
6. Behaviour grouping	No clustering – group by month.	For each day type (weekday and weekend day), obtain 4 clusters using K-means.	For each day type (weekday and weekend day), obtain 3 clusters using K-means, as well as one for no-travel days.
7. Profile generation	Train generative adversarial networks (GANs) to generate realistic profiles for each behaviour group.		
8. Scaling factor transition characterisation	Using 50 discrete time intervals for each day type transition, obtain the discrete transition probabilities between subsequent days.		
9. Behaviour cluster transition characterisation	No clustering.	Compute the transition probabilities for each cluster type and day type transition based on the real datasets.	

### Anonymised data selection and import

Anonymised disaggregated load and PV generation profiles are obtained from the Customer-Led Network Revolution (CLNR), a UK-based smart grid demonstration project [1,2], which collected data from 13,000 customers between 2011 and 2014. PV sources have nominal capacities between 1.35 and 2.02 kWp.

We use anonymised mobility data from the National Travel Survey (NTS) [3] from 105,912 Great Britain households between 2002 and 2020. The NTS surveys the general population's travel patterns and does not focus on EVs – we have selected this dataset rather than an EV trial data, as this offers a less biased view into the general population's travel patterns thanks to both the larger volume of data available, and because the self-selected EV early trial participants may not be representative of patterns once EVs become widely adopted. We assumed that internal combustion engine (ICE) car travel patterns can be substituted for those of EVs, within battery constraints [22].

To overcome memory issues as well as limit computational time, the datasets are broken down into n segments, without interrupting data for single homes. Data size reduction steps such as data filtering and granularity adjustments are conducted first before merging the different streams.

A limitation of these datasets is that behaviour and load profiles may have evolved since the date of collection. For example, the use of incandescent rather than LED lights was more common historically [23], and work patterns have evolved [24]. Moreover, the datasets were collected in the UK, and may not be representative of other countries [25]. However, the methodology proposed could be used with other datasets for different contexts. Finally, the dataset does not provide information on the breakdown of the household loads. While the share of households using electric heating [26] and possessing at-home EV chargers [27] was low at the time of the data collection (2011–2014), it is possible that some heating and transport electrical loads may already be in the source data. There may therefore be a risk of double counting these loads if they are also modelled separately.

### Data selection and filtering

Firstly, the measurements of interests are selected. In the case of the NTS data, only household car trips are conserved, and only homes that can be classified as urban and rural are used. This is because the household type is needed to infer driving type and convert trips into electricity use at a later stage. Moreover, we remove trips above maximum user-defined hourly and daily energy demand, which would not be feasible with an electric car.

Then, the start and end times for data validity for each home are enforced and data beyond valid ranges discarded. Data validity ranges are characterised by the start of valid time, the end of valid time, and the duration of valid time. If one of these is missing, it can be inferred. If two or more of these pieces of information are missing, the validity of data cannot be confirmed, and it is discarded.

### Conversion to relevant daily profiles

Sequences of subsequent data points for single homes are converted to the required resolution (e.g., hourly), and split into individual days.

In the case of CLNR data, this time granularity must be lower than that of the original data, e.g., one minute for PV generation and 30 minutes for household loads. Incomplete days with more than one consecutive data point missing are discarded.

In the case of the NTS travel data, lists of trips are converted to daily profiles of distance travelled. The at home-availability of the vehicles is then inferred from the recorded journeys' origin and destination. Equivalent EV energy consumption profiles are obtained using representative consumption factors from a tank-to-wheel model proposed in [22], dependant on travel speed and type (rural, urban, motorway). Motorway travel is assumed for trips larger than 10 miles.

### Missing data interpolation

For days with missing data points due to data recording or communication issues during the data collection, the options are either to interpolate the missing data points, or to discard the day of data entirely [28]. In this work, we discard days containing series of two or more subsequent data points missing, and we interpolate single missing data points. Continuous data profiles can be generated by filling in the missing data periods with imputed data [28]. This is so that we can increase the number of available full days of available data, making the HEDGE tool is more representative of a wealth of real-life behaviours, while not compromising data quality.

To fill in single missing data points, we test the following options:

1. Linearly interpolate between time steps before and after
2. Replace with the datapoint at the same time the day before or after (whichever has the lowest sum of squares of differences between the previous and subsequent point on the current day)
3. Replace with the datapoint at the same time one or two days before or after (whichever has the lowest sum of squares of differences between the previous and subsequent point on the current day)
4. Replace with the datapoint at the same time one day or week before or after (whichever has the lowest sum of squares of differences between the previous and subsequent point on the current day)

**Fig. 3:**
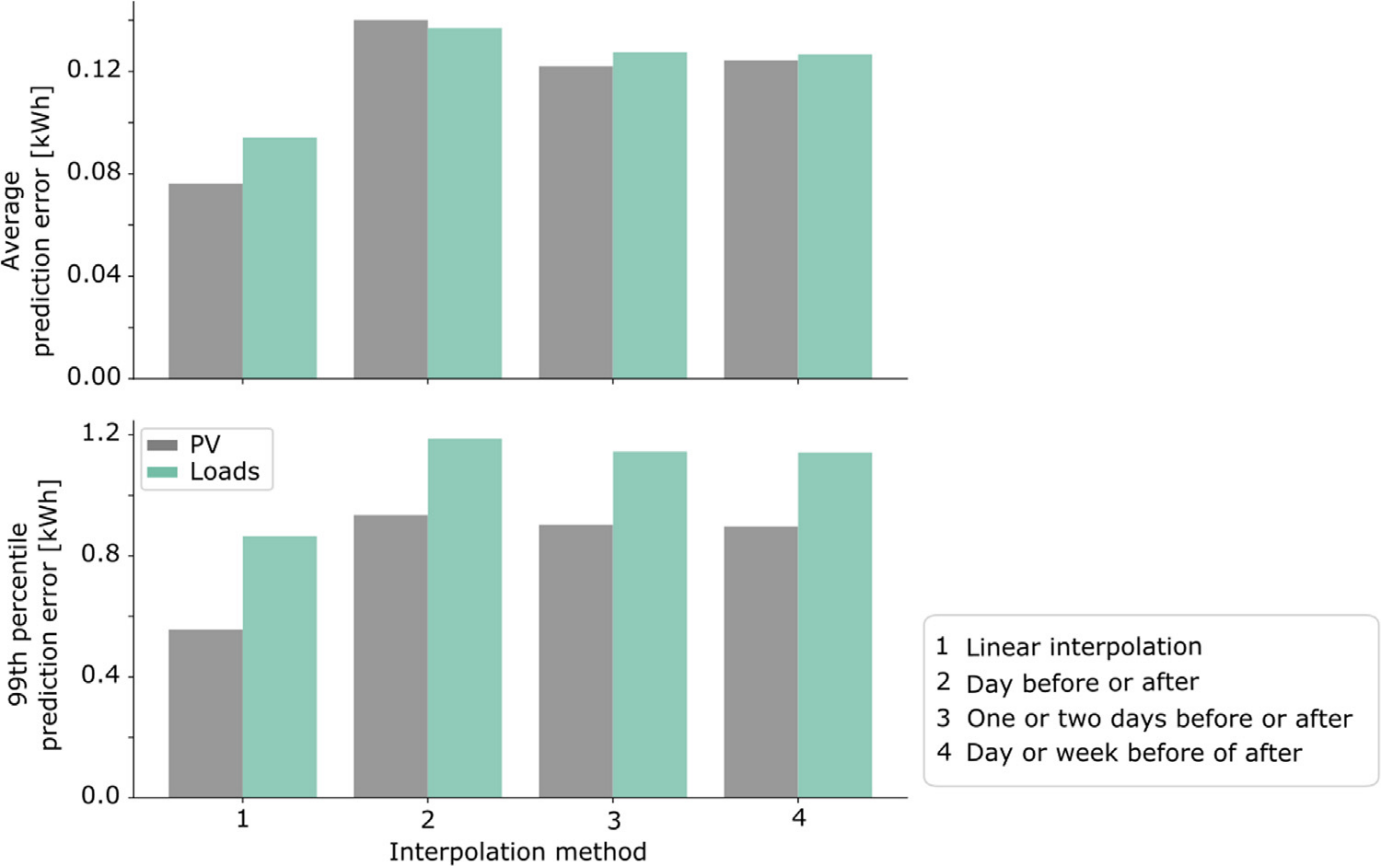
Comparison of interpolation methods.

As shown in Fig. 3, linearly interpolating results in the lowest average and 99th percentile. We therefore use this method to fill in single missing data points.

### Normalisation

Normalisation is performed ahead of profile clustering and GAN training. Each daily profiles for energy generation and consumption are normalised such that $\sum_{t=0}^{24}x\left(t\right)=1$, and the corresponding scaling factors are recorded.

These profiles can then be scaled up consistently to match the expected total energy generation/consumption over a day for a given household by the generation tool, as further described in Sections 2.8 and 3.

### Behaviour clustering

For behaviour-dependant profiles, namely household loads and EV patterns, the normalised profiles are grouped into clusters based on behavioural patterns for both weekday and weekend days. This clustering facilitates the creation of a repository of normalised profiles for each cluster group. This collection of profiles can subsequently serve as the foundation for training GANs to generate profiles representative of each cluster. When using HEDGE, different homes will have different likelihood of belonging to each behaviour group, and profiles can be generated accordingly to maintain consistency.

We use K-means, minimising the within-cluster sum-of-squares [29] in four clusters for both weekday and weekend data (with one for no travel). The features used for load profiles clustering are normalised peak magnitude and time, and normalised values over critical time windows^4^, and those for travel are normalised values between 6 am and 10 pm. PV profiles were grouped per month. The user can define the number of clusters as an input.

**Fig. 4:**
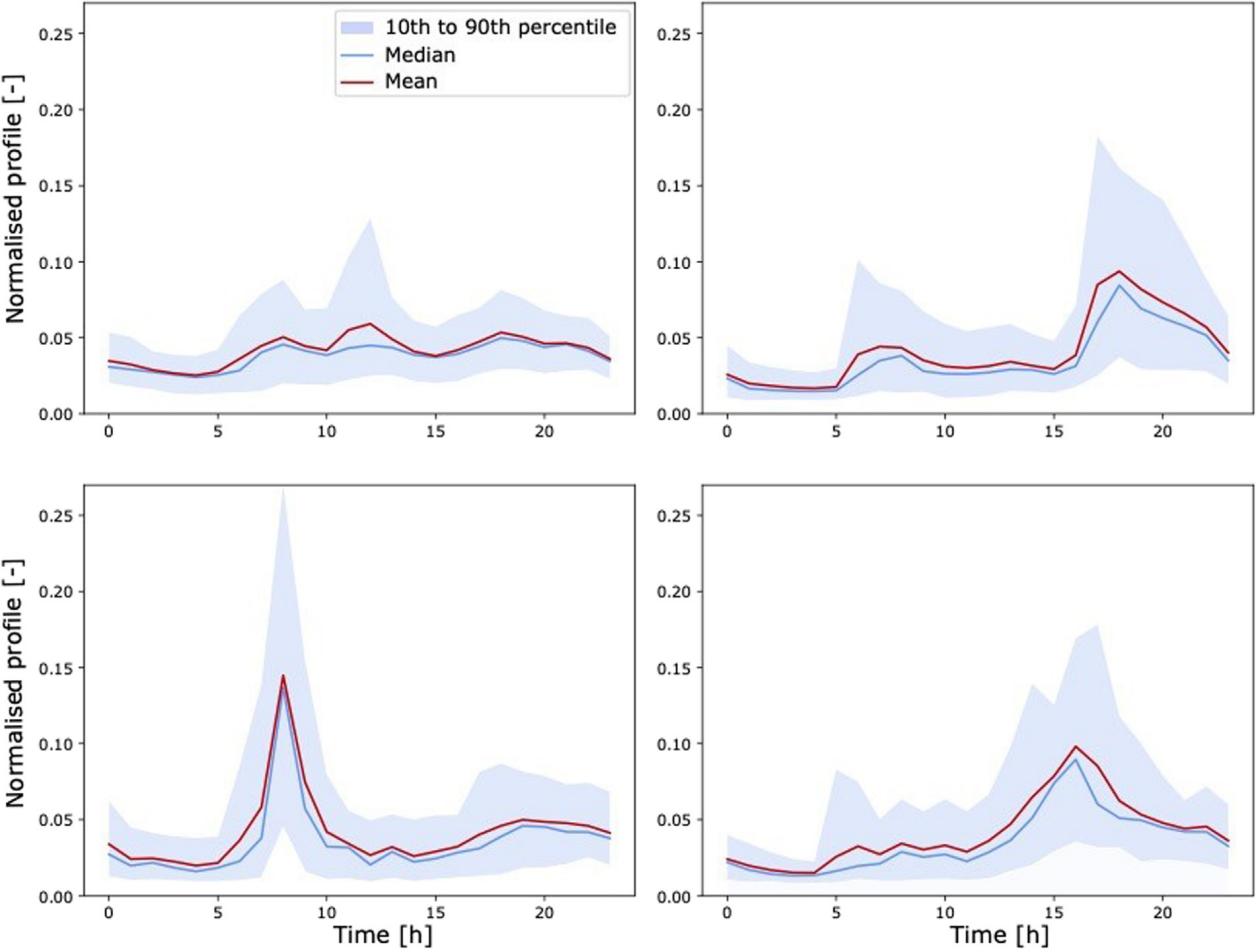
Four behaviour clusters for weekday household electricity consumption normalised profiles.

**Fig. 5:**
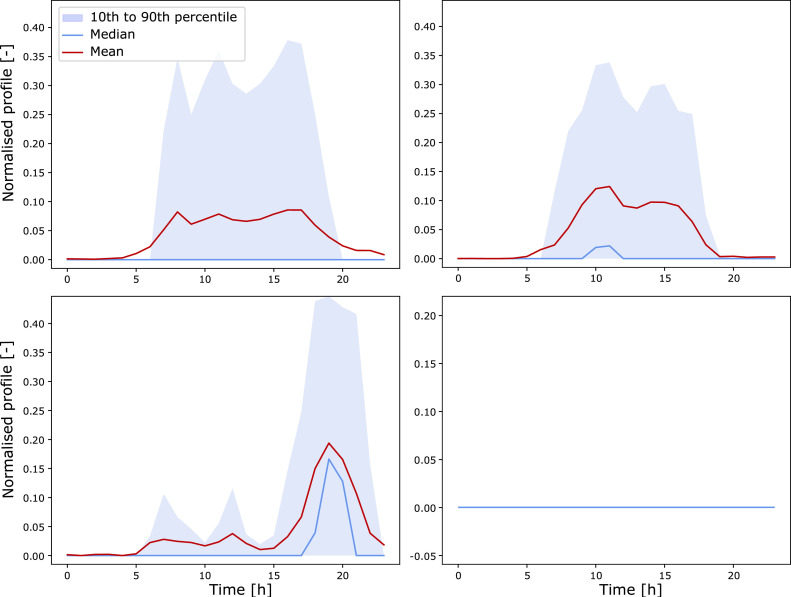
Four behaviour clusters for weekday EV consumption normalised profiles. The fourth cluster corresponds to days with no travel.

As an example, the weekday behaviour clusters for household load and EV consumption are illustrated in Fig. 4, Fig. 5.

### Profiles generation

Neural networks are then trained to generate populations of realistic normalised profiles corresponding to each behaviour cluster and day type. Pre-training neural network weights means that researchers and practitioners do not need to download large databases (here, the raw databases that had to be downloaded were of size 40.12 GB) and run time- and computational resource-hungry data preparation and training steps. They only need to download the pre-trained weights (files of size 125 kB) and perform a feed-forward to generate realistic training and testing data using HEDGE.

**Fig. 6:**
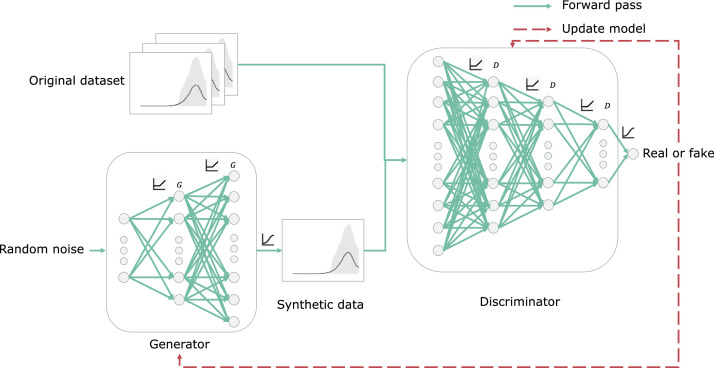
Generative adversarial networks architecture for a given behaviour cluster and day type.

As illustrated in Fig. 6, GANs [30] consist of two simultaneously trained models. The generative model G takes as input a random noise vector z and produces fake data $x_{synthetic}=G\left(z\right)$, aiming to fool the discriminator into thinking they are from the original dataset $x_{real}$. The discriminator model D takes as input data x and produces a probability score D(x)∈[0,1] that indicates the likelihood that x is real data.

Each network aims to minimise the following losses during training:

• The discriminator D aims to maximise the probability of correctly discriminating between the real data and the fake data generated by the generator network G, by minimising the binary cross-entropy between the real (1) and fake (0) labels and the probabilities assigned by the discriminator:

$l_{D}=-E_{x_{\text{real}}}\left[\log D\left(x_{real}\right)\right]-E_{z}\left[\log\left(1-DG\left(z\right)\right)\right]$

• The generator loss is calculated from the discriminator's classification – It gets rewarded if it successfully fools the discriminator and gets penalised otherwise. The loss function aims to minimise the binary cross-entropy between the fake labels and the probabilities assigned to the fake generated data by the discriminator:

$l_{G}=-\;E_{z}\left[\log\left(D(G\left(z\right)\right)\right]$

To generate realistic populations of synthetic profiles, we use the following additional parameters and algorithm configurations:

• We exponentially decay the learning rate to avoid oscillation and to obtain faster convergence [31], so that the learning rate at each epoch is:

$\alpha_{epoch}=\alpha_{0}{\left(\frac{\alpha_{\text{end}}}{\alpha_{0}}\right)}^{\frac{epoch}{n_{\text{epochs}}}}$

• We enforce the positivity of the generator's output by using the sigmoid activation function [32] on the last layer on the generator network:

$\sigma \left(x\right)=\frac{1}{1+e^{-x}}$

• We employ dropout layers [33] within the neural network architectures to improve the performance of the models. This prevents overfitting to the training data by randomly dropping out (setting to zero) some of the outputs of the neurons during training, with probability $p_{G}$ for the generator and $p_{D}$ for the discriminator, effectively removing them from the network for that iteration. By doing this, the network becomes less sensitive to the specific weights of individual neurons and is forced to learn more robust features that are shared across multiple neurons.

Moreover, we further propose the following:

• We generate a population of profiles i∈{1,…,n} at each forward, pass, rather than one profile. This is to ensure that the GAN generates variability within one population that is realistic, rather than converging towards one realistic profile.
• We add an exponentially decaying noise to the exploration, to improve the efficiency and effectiveness of learning by encouraging exploration, avoiding overfitting oscillation, and obtain faster convergence. The decay helps balance the exploration and exploitation trade-off over time. The noise at each epoch is thus:

$\epsilon_{epoch}=\epsilon_{0}{\left(\frac{\epsilon_{\text{end}}}{\epsilon_{0}}\right)}^{\frac{epoch}{n_{\text{epochs}}}}$

• We add a penalty to the generator's loss if the sum of the generated normalised profiles diverges from 1:

$l_{1}=W_{1}{\left(\frac{\sum_{i}\sum_{t}x_{i}^{t}}{n}-1\right)}^{2}$

• We add a penalty to the generator's loss if the 10th, 25th, 50th, 75th and 90th percentiles and the mean over the whole generated population for each time step t varies from the original dataset for each time step:

$l_{2}=W_{2}\sum_{k\;\in \;\left\{10^{th},25^{th},\;50^{th},\;75^{th},90^{th},\;mean\right\}}\sum_{t}{\left(x_{k}^{t}-x_{real}^{t}\right)}^{2}$
And $l_{G}^{\prime }=l_{G}+l_{1}+l_{2}$

Training parameter values are tabulated in Table 2.

**Table 2:** Generative adversarial network training parameters.

Initial noise $\epsilon_{0}$	1	Batch size m	100
End noise $\epsilon_{\text{end}}$	1e-4	Number of epochs $n_{\text{epochs}}$	200
Initial learning rate $\alpha_{0}$	1e-2	Number of profiles in generated population n	50
End learning rate $\alpha_{\text{end}}$	1e-3	Discriminator dropout probability $p_{D}$	0.3
Normalised profiles loss weight $W_{1}$	0.1	Generator dropout probability $p_{G}$	0.15
Percentile distance loss weight $W_{2}$	100		

### Assessment of generative adversarial networks

Assessing the performance of GANs can be challenging, especially for GANs generating time-series data, which is a more nascent field of study relative to the computer vision domain. A combination of both qualitative and quantitative assessments is recommended [34].

**Fig. 7:**
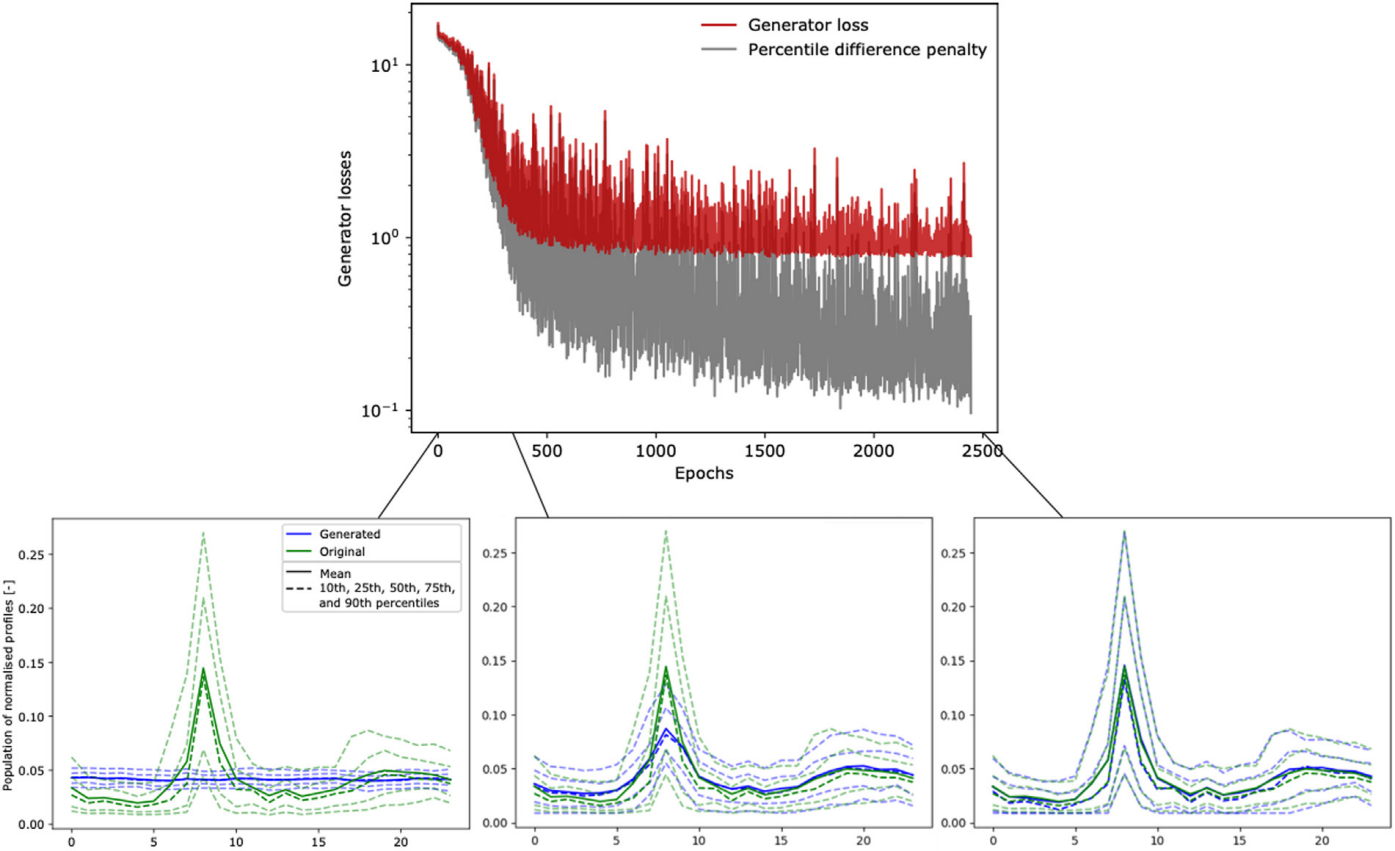
Example of generated populations of 50 household load normalised profiles against the distribution of the original dataset throughout the GAN training.

Firstly, we therefore perform a qualitative visual assessment of the profiles generated by the GAN. An example of a generated population of 50 household load profiles throughout the training is presented in Fig. 7. While the profiles generated before the training starts do not match the target distribution, the population of profiles that is generated at the end of the training visually matches the target population in terms both of mean and in terms of the distribution and variability of the population of profiles throughout the day. This shows that the generated profiles are diverse enough, as samples are distributed to cover the real data.

Secondly, we perform a quantitative evaluation, by adopting the “Train on Synthetic, Test on Real” (TSTR) framework proposed in [35] to evaluate the output of a GAN. This framework tests the usefulness of the GANs, by assessing the extent to which the generated data maintains the predictive attributes of the original. The testing sequence is as follows:

1. Split the real dataset into a training (80% of the data) and a testing (20%) dataset.
2. Train the GANs using the training dataset.
3. Generate synthetic data with the GANs.
4. Train a model using the synthetic data – Here, we train a classifier which aims at predicting which cluster a population of data profiles belongs to.
5. Test the classifier model using the held-out testing data. By determining the classifier's quality, this evaluation method, in turn, thus aims at assessing the quality of the generated data in being used for real applications.

Similar to the TSTR method, we also consider the reverse case, called “Train on Real, Test on Synthetic” (TRTS). Steps 1, 2 and 3 are identical, and steps 4 and 5 are interchanged as:

4. Train the classifier using the held-out testing data.
5. Test the classifier model using the synthetic data.

**Fig. 8:**
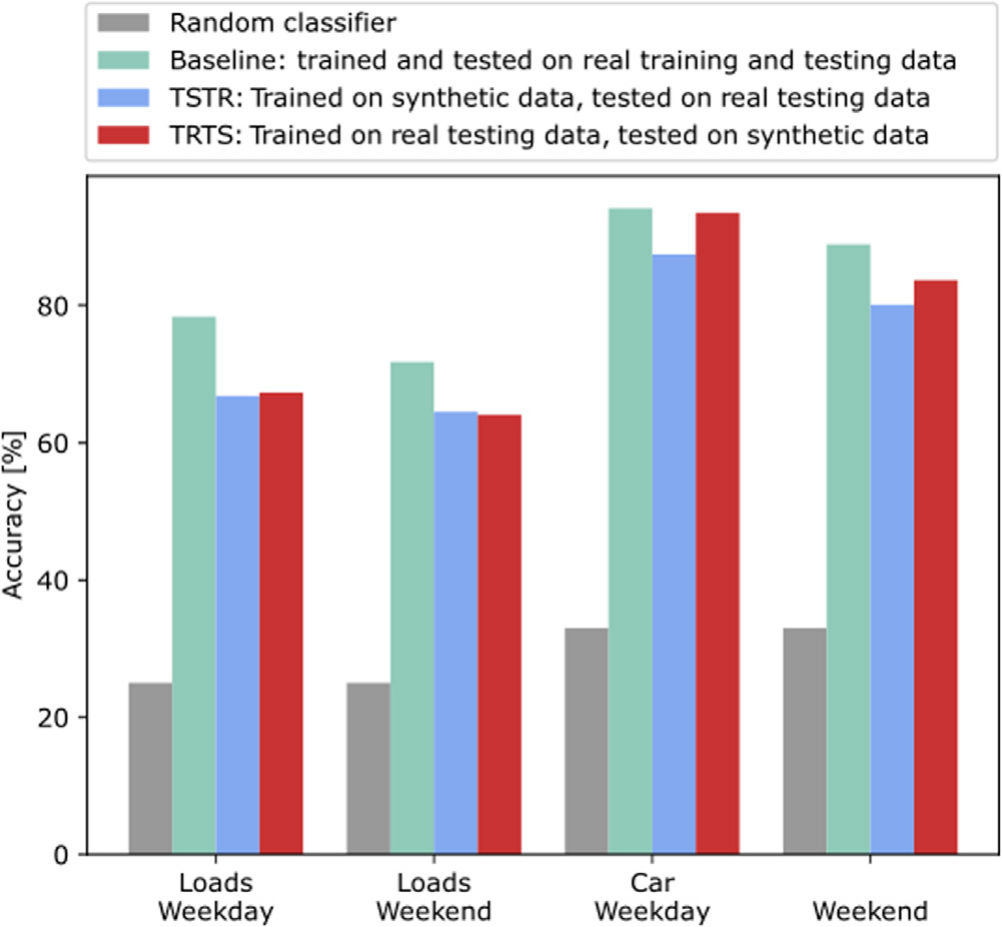
“Train on Synthetic, Test on Real” and “Train on Real, Test on Synthetic” accuracy scores using the trained GANs relative to random and baseline classifiers. Average accuracy over 10 repetitions.

The performance of the classifiers in the TSTR and TRTS experiments presented in Fig. 8 shows that the synthetic data generated by the trained GANs is useful for subsequent applications.

### Scaling factor transition characterisation

The unit-less normalised profiles generated by the trained GAN networks must then be scaled by a scaling factor consistent with a given home to produce profiles in energy units.

We use transition matrices to model the probability of transitioning from one scaling factor $f_{t}$ to the next one $f_{t+1}$ in subsequent days. Using these matrices allows the data generator to scale subsequent days of data consistently, with variability around self-correlation that matches that of real-life observed patterns for each data type and weekday type (weekday or weekend day). The space of possible scaling factors is discretised into m intervals. The probability of transitioning from discrete factor intervals i and j is then:

$$p_{i,\;j}=\frac{n_{i,j}}{\sum_{k}n_{i,k}}$$

Where $n_{i,j}$ is the number of times that a transition between intervals i and j was recorded in subsequent days of data available.

As the probability of scaling factors is not evenly distributed between the minimum and maximum factors, we adopt a non-uniform discretisation approach based on percentile intervals, with finer data intervals for more common, lower scaling factors, and wider intervals for less common ones. This ensures that we retain granularity and information for more common lower factors. Furthermore, we use the 2D piecewise linear interpolation to fill in gaps in probability intervals, while ensuring the sum of probabilities for the next day always equals one.

**Fig. 9:**
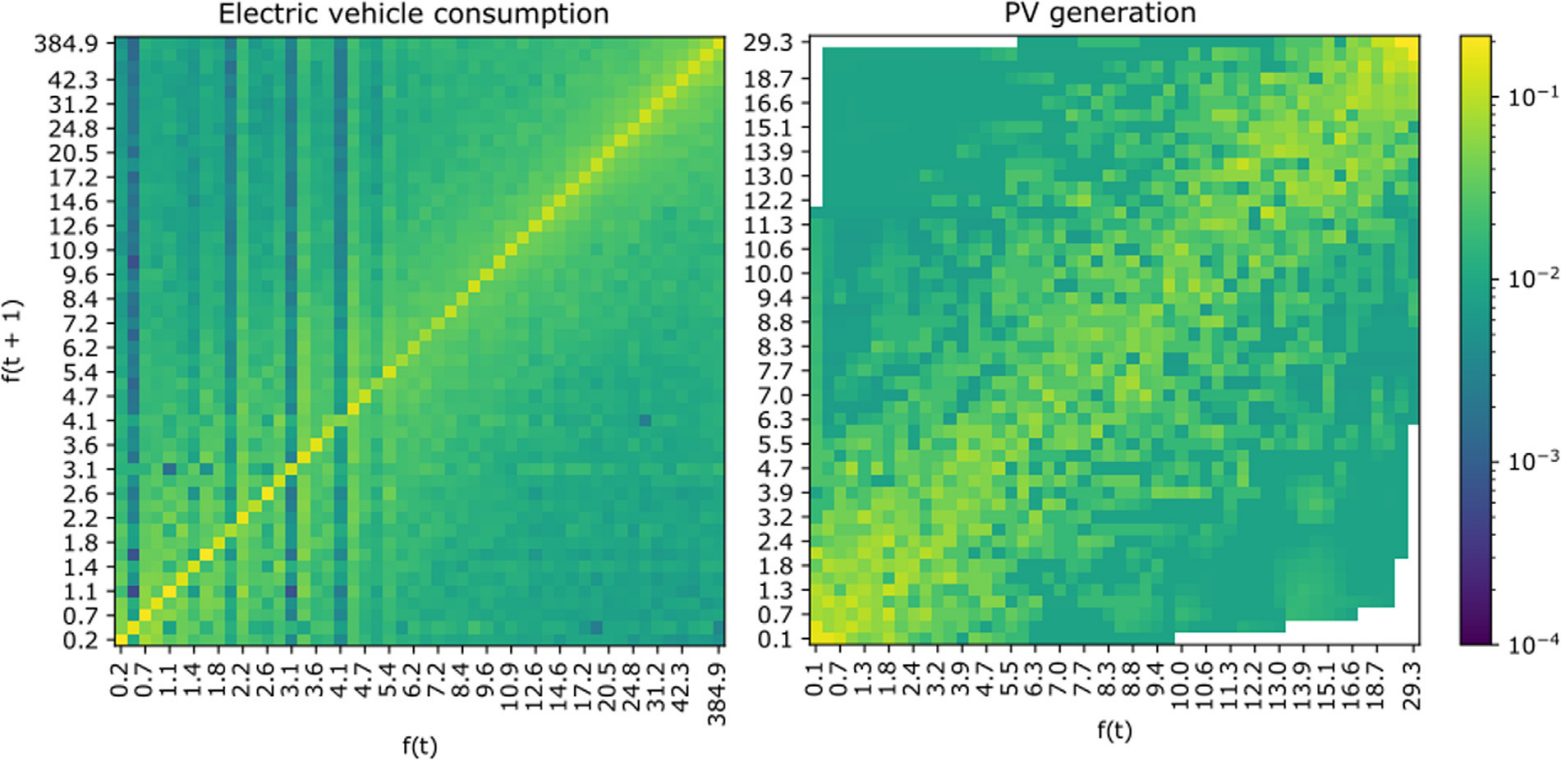
Transition probability matrices between profile scaling factors in subsequent days (*m* = 50 intervals).

Matrices of scaling factors transition probabilities $P_{f}\left(f_{t+1}|f_{t},\;c_{t},\;c_{t+1}\right)$ are illustrated in Fig. 9.

### Behaviour cluster transition characterisation

In the case of behaviour-dependant data (household loads, EV patterns), we similarly characterise the probabilities $P_{c}\left(c_{t+1}|c_{t},\;d_{t},\;d_{t+1}\right)$, of transitioning from one behaviour cluster to another in subsequent days for each day type transition ($d_{t}$ being weekday or weekend day), so that profiles can be generated using the adequately trained GAN networks. Variations in generated behaviour thus match real-life patterns for each new day.

## Home electricity data generator (HEDGE) tool

From the data processing described in Section 2, we obtain the following inputs for the Home Energy Data Generator (HEDGE):

a. Behaviour cluster transition matrices $P_{c}$
b. Normalised profiles generator (per data type, day type and behaviour cluster)
c. Scaling factors transition matrices $P_{f}$

Behaviour clusters (e.g. based on Fig. 4, to which cluster is the home closest on the day preceding the start of data generation?) and scaling factors (i.e. what is the total energy used in the day preceding the first day of data generated?) are first initialised for each home. These do not have to be real-time detailed data, but rather aim to give an indication of the type of home considered. They are automatically selected in HEDGE to match their distribution in the original dataset if not specified by the user. Then, a Markov chain mechanism uses these to generate profiles for successive days, consistent across both scaling factors and behaviour clusters. The probabilistic Markov chain transition rules are:

1. For behaviour-dependant data types, select behaviour cluster $c_{t}$ based on the behaviour cluster transition matrix $P_{c}\left(c_{t+1}|c_{t},\;d_{t},\;d_{t+1}\right)$, to select the appropriate GAN profile generator.
2. Generate a population of normalised profiles using pre-trained GAN weights for the relevant data type, day type and cluster. Randomly select one of the generated profiles.
3. Scale the profile using a scaling factor according to the probabilities in the scaling factors transition matrices, from discrete distribution $P_{f}\left(f_{t+1}|f_{t},\;c_{t},\;c_{t+1}\right)$

New random, realistic data can thus be generated for each subsequent day of simulation.

## Energy user privacy preservation

The mitigates privacy concerns for experimentation with realistic residential energy data. During the data pre-processing and neural network training phase (Section 2), only anonymised disaggregated data is used from established datasets. In the data generation phase (Section 3), only pre-computed statistics and weights derived from these anonymised datasets are required. Moreover, the generated data does not pertain to any real energy user. Rather, the generated data is synthetic but realistic data that can be used for experimentation.

## Ethics statements

This work abided to MethodsX ethical guidelines and did not involve human subjects, animal experiments, or data collected from social media platforms.

## CRediT authorship contribution statement

**Flora Charbonnier:** Conceptualization, Formal analysis, Methodology, Software, Validation, Writing – original draft, Visualization. **Thomas Morstyn:** Conceptualization, Validation, Writing – review & editing, Supervision. **Malcolm McCulloch:** Resources, Writing – review & editing, Supervision.

## Declaration of competing interest

The authors declare that they have no known competing financial interests or personal relationships that could have appeared to influence the work reported in this paper.

## Data Availability

Data will be made available on request.

## Footnotes

3 Scopus key word search selecting for publications whose title and abstract include at least one mention of each the residential sector (home or residential), of data-driven methods (data-driven, learning, big data, or forecasting), the energy sector (energy, electricity, power, voltage, renewable) and local energy appliances (car, PV, solar, loads, smart, IoT, storage, battery, heating, HVAC, generation, electric vehicle, EV, appliance, demand response, demand-side response, peer-to-peer, consumer, or fridge)

## Footnotes

1 https://github.com/floracharbo/hedge.

2 interquartile range with no or limited overshoot (high confidence).

4 0-7 am, 7-11 am, 11 am-2 pm, 2-5 pm, 5-9 pm, 9-12 pm.
